# Understanding Malicious Accounts in Online Political Discussions: A Multilayer Network Approach

**DOI:** 10.3390/s21062183

**Published:** 2021-03-20

**Authors:** Nhut-Lam Nguyen, Ming-Hung Wang, Yu-Chen Dai, Chyi-Ren Dow

**Affiliations:** Department of Information Engineering and Computer Science, Feng Chia University, Taichung 40724, Taiwan; lam@mail.fcu.edu.tw (N.-L.N.); m0825773@mail.fcu.edu.tw (Y.-C.D.); crdow@mail.fcu.edu.tw (C.-R.D.)

**Keywords:** social media, malicious users, influential users, information manipulation, political propaganda, multilayer network

## Abstract

Online social media platforms play an important role in political communication where users can freely express and exchange their political opinion. Political entities have leveraged social media platforms as essential channels to disseminate information, interact with voters, and even influence public opinion. For this purpose, some organizations may create one or more accounts to join online political discussions. Using these accounts, they could promote candidates and attack competitors. To avoid such misleading speeches and improve the transparency of the online society, spotting such malicious accounts and understanding their behaviors are crucial issues. In this paper, we aim to use network-based analysis to sense influential human-operated malicious accounts who attempt to manipulate public opinion on political discussion forums. To this end, we collected the election-related articles and malicious accounts from the prominent Taiwan discussion forum spanning from 25 May 2018 to 11 January 2020 (the election day). We modeled the discussion network as a multilayer network and used various centrality measures to sense influential malicious accounts not only in a single-layer but also across different layers of the network. Moreover, community analysis was performed to discover prominent communities and their characteristics for each layer of the network. The results demonstrate that our proposed method can successfully identify several influential malicious accounts and prominent communities with apparent behavior differences from others.

## 1. Introduction

Online social media platforms have become popular tools where users can freely share and exchange information as well as express personal views on certain issues. For instance, Twitter allows its users to post their own tweets, share the tweets of others, and express their opinion by “liking” tweets. In politics, social media platforms have been used as a means for political parties and their candidates to disseminate information, monitor public opinion, attract votes, and even attack the opponents [1]. For this purpose, each political organization usually creates an account to formally represent their organization for announcing their statements on social media [2]. On the other hand, some political organizations may also create several accounts for the purpose of guiding and polling public opinion [3]. They publish articles that polish their candidate and criticize the competitors. These malicious accounts could even disrupt the fairness of an election; and if users are not firm, they are easily driven by the information that such accounts generated.

**Figure 1:**
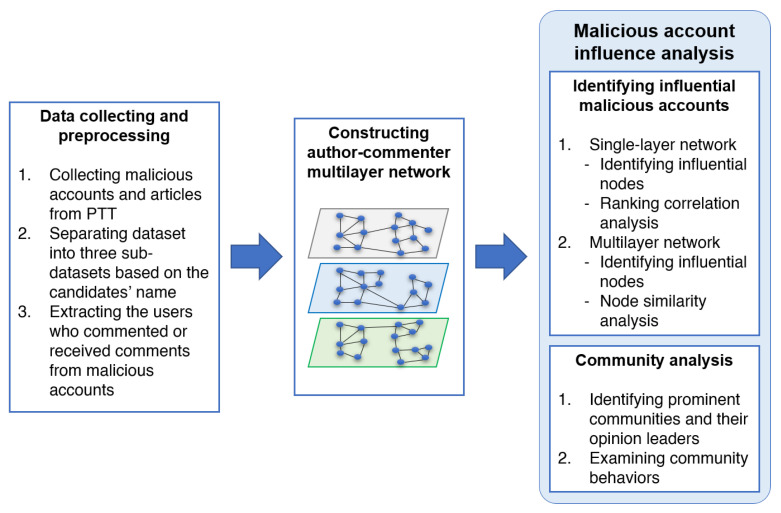
Overview of our approach.

To identify such underneath malicious accounts, an intuitive solution is spotting influential users on social media. This topic has been attracted significant attention from scholars, especially in political discussion forums [4,5]. A social network is usually represented as a graph (single-layer network) where nodes denote the users and edges represent the relationship or interactions among them [6]. However, representing users and their relations as a single-layer network for identifying influential users may result in partial relationship information representation [7]. Multilayer networks, as shown in Figure 1, which consists of a family of graphs, have been successfully used for modeling social networks in previous studies to, for example, detect suspicious behaviours [8], identify influential spreaders [7], and find multiple leaders [9]. In online political discussion forums, a user might be interested in the discussions related to one candidate only, while others may participate in the discussions related to many different candidates. Therefore, by modeling the political discussion networks as a multilayer network where each layer represents the discussions related to one candidate, we can explore the network structure of malicious accounts participating in different political discussions. This approach facilitates us to inspect the interactions and influences of malicious accounts not only in a single layer but also across different layers of the network.

In this paper, we attempt to investigate influential malicious accounts in political discussion forums taking Taiwan as a case study. The dataset used in this study was collected from PTT Bulletin Board System (PTT), the most influential discussion forum in Taiwan. We focus on the following research questions:

- RQ1: Which malicious accounts are the most influential accounts in the discussion networks?
  - RQ1.1: Which malicious accounts are the most influential accounts in Tsai’s, Han’s, and Ko’s discussion networks, respectively?
  - RQ1.2: Which malicious accounts are the most influential accounts in the whole network?
  - RQ1.3: Are the ranks of influential malicious accounts persistent across different networks?
- RQ2: Does the interaction of malicious accounts vary across different networks?
- RQ3: What is the community structure of each network?
  - RQ3.1: Which communities are the prominent communities in each network?
  - RQ3.2: Which malicious accounts are the opinion leaders of each prominent community?
- RQ4: What is the main activity of each prominent community?
  - RQ4.1: Which communities are the most active communities regarding the number of posted articles and the number of given comments?
  - RQ4.2: What are the temporal trends of comments and articles posted by each prominent community during the observation timeframe?

To address the above research questions, we proposed an approach to identify influential malicious accounts (sockpuppet accounts belong to a specific person or group) verified by the administrator of PTT. Unlike in previous works, in this study we represent the political discussion as a multilayer network in which each layer visualizes the discussion related to a particular candidate. We apply various centrality measures to investigate influential malicious accounts not only in a single layer but also across all layers of the network. The interlayer correlations are calculated to verify whether the influence of malicious accounts is permanent across different layers. We further compute the node similarity to assess the interaction consistency of node across different layers. Finally, community detection algorithms are deployed to extract the communities of each layer, from which we can understand the main activities as well as the influence of influential malicious accounts in these communities.

The rest of this paper is organized as follows. Section 2 discusses the related work. The proposed approach is presented in Section 3. We illustrate the experimental results and analysis in Section 4. Finally, we present the issues to be addressed in the future and conclude our work in Section 5.

## 2. Related Work

Studying the impacts of malicious users on electoral campaigns has been an attractive research topic in the past few years [10,11,12]. Accounts created for the purpose of manipulating public opinion, polling, and attacking opposing candidates in an electoral campaign are considered to be political bots, trolls, sockpuppets, and political astroturfing users. The vital question is whether malicious users have negative or positive effects on the democratic political discussions as well as the election outcomes. In [10], by investigating more than 20 thousand million tweets related to the 2016 U.S. Presidential election, the authors concluded that the tweets created by social bots have negative effects on the discussion related to the election. The polarity and sentiment of the discussion network were clearly influenced by social bots as removing them from the network resulted in decreasing the discussion’s sentiment scores [11]. Social bots can indeed cause a change in public opinion [12].

Unlike social bots, accounts that are operated by computer algorithms, malicious users such as sockpuppets and political astroturfing accounts are controlled by human beings. Spotting these accounts is often more difficult compared to bots, although bots these days try to mimic human behavior in order to conceal their existence. Such malicious accounts may act independently or in groups with dedicated political stances to specific political entities, and they are probably recruited by political entities to influence public opinion [13]. A new type of malicious accounts that manipulates public opinion in electoral campaigns is called “cyber army” [14]. These malicious accounts are operated by humans to support a certain candidate and denounce the opposing ones. Studying the behavior of malicious accounts in electoral campaigns taking Taiwan as a case study has been conducted in previous studies [14,15,16]. These studies revealed some important findings regarding the activities of malicious accounts such as publishing an unusual number of articles, giving a significant number of comments to support their posts, and less tendency to give negative ratings to the articles compared to normal users.

Influential users or opinion leaders play an important role in political discussion [17]. These accounts have the ability to influence the attitude of other users, shape political opinion, or even sway the election outcomes [18]. The purpose of influential users is to rapidly disseminate information such as news topics to other users in a discussion forum as much as possible. Cha et al. [19] investigated influential users on Twitter and found that the indegree (the number of users following a particular user) is related to their popularity. Feng [20] introduced five types of central users including conversation starter, influencer, active engager, network builder, and information bridge. Among them, conversation starters who have a significant number of indegree links but a few or none outdegree links, and influencers who have numerous indegree links with a few outdegree links, are considered to be opinion leaders and information sources in long-term discussions on Twitter [21].

Identifying the most influential users who play an important role in spreading information on social media platforms has received much attention from various domains such as sports [22], healthcare [23], and, more importantly, political discussion [24]. Graphs have been used as a powerful tool for identifying influential users in social networks. Dubois and Gaffney [24] collected tweets related to two Canada’s largest political Twitter communities (Conservative Party of Canada and New Democratic Party of Canada) and constructed a follower graph for ranking the most influential users using centrality measures such as indegree and eigenvector. The authors found that different metrics rank influential users differently and the indegree metric works well on identifying influential users who are highly visible such as journalists and politicians. Similarly, Benigni et al. [25] collected the ALT16 (alt-right community) dataset and created a directed mention graph to find influential users using degree centrality, PageRank, and coreness [26] metrics. Zayer and Gunes [27] studied the visual impairment awareness campaigns on Twitter. One of the main goals of this work was to identify the key players who played a major role in information dissemination during the campaigns. The authors considered the key players as those whose tweets were retweeted by a significant number of other users. They created the retweet network and ranked the players according to their indegree centrality. Ranking the users on Twitter by the number of followers and PageRank yields similar results, but they differed from the ranking by retweets [28].

## 3. Proposed Approach

In this section, we describe our approach for positioning and analyzing influential malicious accounts and their communities on the political discussion forum in Taiwan, which is illustrated in Figure 1. The four major steps are listed as follows:

1. We collect the dataset from PTT, the most influential discussion forum in Taiwan, and the malicious user list announced by the PTT official.
2. We preprocess the collected dataset and extract the users who participated in malicious accounts’ discussions.
3. We model the online political discussions as a multilayer network and identify influential nodes as well as their communities using various centrality measures.
4. We conduct the experiments and analyze the experimental results from various aspects.

### 3.1. PTT

PTT is the most influential social discussion platform in Taiwan. According to Alexa’s traffic analysis tools, PTT is ranked #20 and #996 in Taiwan and in global internet traffic and engagement, respectively (https://www.alexa.com/siteinfo/ptt.cc#section_traffic (accessed on 9 February 2021)). This forum has over 1.5 million users with more than 20 thousand boards discussing a variety of topics. PTT has been considered to be a major online social platform for studying malicious behaviors in political discussion in Taiwan in previous studies [14,16]. Like Reddit, a popular news aggregator and web content rating, the articles posted on PTT are categorized into discussion boards by their topics. The boards cover a variety of topics such as politics, jokes, and movies. On PTT, users can have interactions by publishing/commenting/rating.

**Figure 2:**
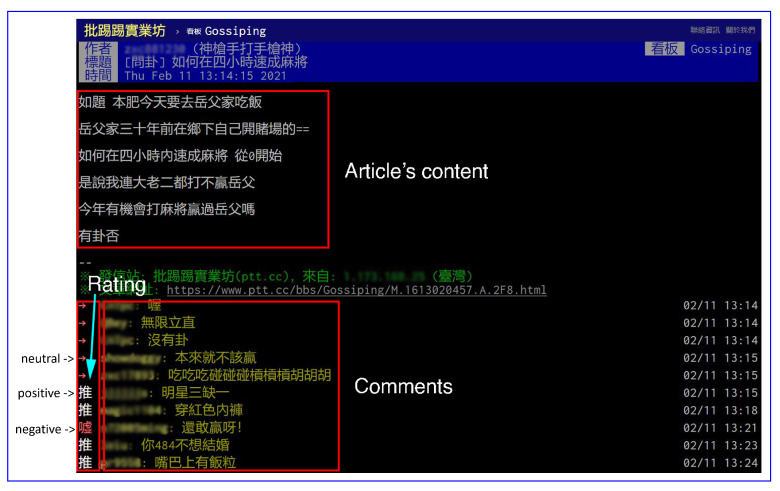
Screenshot of an article posted on PTT (https://www.ptt.cc/bbs/Gossiping/M.1613020457.A.2F8.html (accessed on 11 February 2021)).

Figure 2 shows an example of the article that is posted on PTT. As shown in the figure, each article comprises two parts: the content of the article and the comments given to the article. The users in the forum can give more than one comment on an article and express their emotion by ratings. The “推” (push) rating indicates that the commenter has a positive attitude towards the article. By contrast, the “噓”(boo) rating means that the commenter dislikes the article. The rating “→” indicates that the commenter has a neutral attitude towards the article.

### 3.2. Data Collection

The main purpose of this study is to positioning influential malicious accounts and their activities during the electoral campaign; therefore, we only considered the “Gossiping” board (https://www.ptt.cc/bbs/Gossiping/index.html (accessed on 15 September 2020)) which focuses on political issues. We first collected all articles which contain the names of three major politicians in Taiwan: the Taipei City Mayor Ko Wen-Je, the Kaohsiung City Mayor Han Kuo-Yu, and the incumbent president Tsai Ing-Wen from 24 May 2018 to 11 January 2020 (the election day). Each entry in the dataset consists of the following information:

- Article metadata: Each article has its corresponding metadata, which includes the article ’s ID and the submission time.
  Article content: Each article has its title and the main content.
  Author information: Author information of an article includes the author nickname, author ID, and IP address.
  User comment and rating: An article may have more than one comment. Each comment contains the textual part, the user ID of the commenter, the comment timestamp, and the rating. The rating can be positive/negative/neutral rating.

We then grouped the crawled articles into three sub-datasets based on the name of the candidate mentioned in the article. Malicious accounts, which are owned by specific users or groups, were obtained from the administration of PTT. The malicious account list verified by the PTT official is announced on the board called “ID_Multi” (https://www.ptt.cc/bbs/ID_Multi (accessed on 15 September 2020)). According to the recent regulations of multiple account management on PTT, a person can have a maximum of five accounts and the profile information of those accounts must be the same. If the administrators found a large number of accounts that are suspected to be the same person but declared profile information are different, they will conduct a manual investigation on them to verify if they are sockpuppets. We crawled this board and manually extracted the accounts in each judgment. We filtered the crawled data so that it only contains articles or comments posted by malicious accounts. The summary of our dataset is shown in Table 1. Please note that the statistical values shown in the table are of malicious accounts.

### 3.3. Author-Commenter Multilayer Network

A multilayer social network can be defined as a family of multiple layers, and each of which represents a type of user interaction. A layer in a multilayer social network is a directed/undirected, weighted/unweighted graph, where nodes represent users and links indicate the relationship between them. For instance, in [9], Twitter was modeled as a multilayer social network of follower, retweet, and mention layers. In this network, each Twitter account was represented as a node and the link between two arbitrary nodes denoted their relationship. Inspired by the success of modeling user interactions on online social networks as multilayer social networks, we constructed an author-commenter multilayer network (ACMN) to distinguish the interaction between malicious accounts as well as their behaviors in the discussions related to different candidates.

The ACMN is a multilayer layer network M of three layers $M=\left\{G^{T},G^{H},G^{K}\right\}$, and each layer is a directed, weighted graph representing the interaction between commenters and authors for a particular candidate. More specifically, $G^{T}$, $G^{H}$, and $G^{K}$ represent the interaction between malicious accounts as well as the interaction between them and legitimate accounts, which they have given comments to or received comments from, in the discussions related to candidates Tsai, Han, and Ko, respectively. The layer l∈M is represented by $G^{l}\left(V^{l},E^{l}\right)$, where $V^{l}$ and $E^{l}$ are sets of nodes and links. A node in a layer is a malicious account or legitimate account which participates in malicious accounts’ discussions. There will be a link from account $S_{1}$ to account $S_{2}$ if $S_{1}$ has commented at least one time on the articles posted by $S_{2}$, and the weight of the link is the total number of comments that $S_{1}$ had given to articles posted by $S_{2}$. Please note that nodes in a layer may not appear in other layers as some users tend to comment or post articles related to their focusing candidate, and they might not be interested in commenting or posting articles related to other candidates.

**Figure 3:**
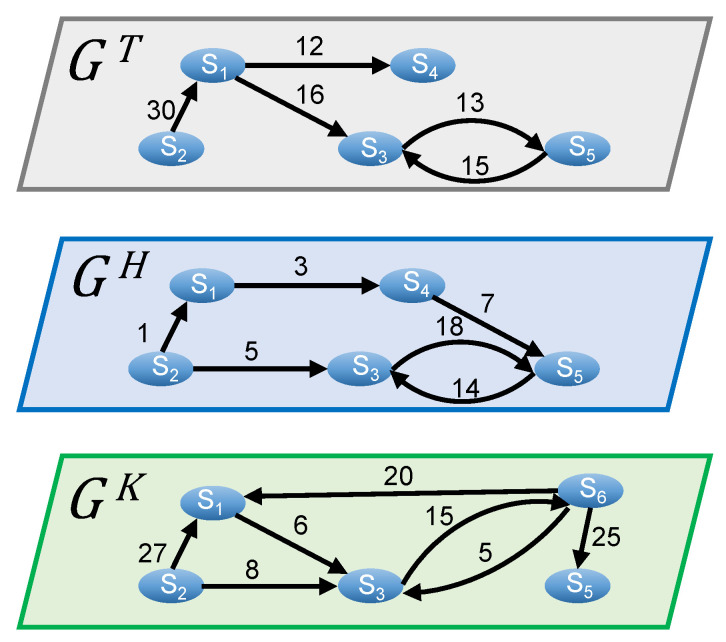
An example of the ACMN.

Figure 3 illustrates an example of the ACMN comprising of three layers $G^{T}$, $G^{H}$, and $G^{K}$, each of which represents the discussions related to Tsai, Han, and Ko, respectively. It can be seen from the figure that the interaction between the same users may differ across different layers. For instance, in layers $G^{T}$ and $G^{H}$, accounts $S_{3}$ and $S_{5}$ have given many comments on the articles posted by each other; however, there is no interaction between these two accounts in layer $G^{K}$. In addition, account $S_{6}$, who only appears in layer $G^{K}$, gives many comments on the articles posted by almost all other users in this layer. This may imply that this account is only interested in the discussion related to Ko. A single-layer network for representing the whole discussion network cannot capture such characteristics. Therefore, by constructing a multilayer network comprising of three graphs where each of them represents the discussion related to one candidate, we can explore the user interactions related to the three candidates more precisely.

### 3.4. Influential Malicious Accounts (RQ1)

To answer RQ1, we first identify the influential malicious accounts for each layer of the ACMN based on the indegree, outdegree, and PageRank. For finding the influential malicious accounts over different layers of the ACMN, we next rank the malicious accounts according to their cross-indegree, cross-outdegree, and multiplex PageRank. Finally, we investigate the influence of malicious accounts across different layers of the network using Spearman’s rank correlation coefficient.

#### 3.4.1. Identifying Influential Malicious Accounts (RQ1.1, RQ1.2)

Influential users attempt to influence the attitudes of other users by spreading their posts and comments. In our work, we are interested in finding influential malicious users who have the following characteristics: (1) receiving comments from many different users, (2) giving comments to articles posted by many different users, and (3) receiving comments from those who get a significant number of comments from many different users. There are various network measures for ranking influential users in social networks such as centrality measures and prestige measures [29]. The users who receive a significant number of comments from different other users are considered to be conversation starter users, and the indegree measure is an effective measure to quantify these users. Similarly, the users who give comments to plenty of different users are referred to as the active engager users. Such users can be effectively identified by using the outdegree metric. An empirical study conducted in [21] showed that the users with high indegree can be considered to be opinion leaders in long-time discussions. In [30], the authors used weighted indegree to identify the influential nodes of the network of people tweeting. In this paper, the weighted indegree of a node is corresponding to the number of mentions that a user has received. In our study, we consider that a user is more influential if he/she receives attention from or gives comments to many different other users; thus, the weighted indegree is not suitable for us. PageRank, a well-known algorithm for ranking web pages, has been widely used as a centrality measure for identifying influential users in social networks  [19,31,32]. As the PageRank of a node makes contributions to the nodes that it points to; therefore, it is appropriate to rank the users with respect to the users who point to them. From the above reasons, in our work, three popular network measures including indegree, outdegree, and PageRank are used to find the influential malicious accounts in our dataset. These metrics have been effectively used for ranking influential users in prior studies [21,24,27,32]. In addition, as the ACMN is a multilayer network, such metrics cannot be directly applied to the network as they only work with a single-layer network. To cope with this problem, we adopted cross-layer metrics, i.e., cross-layer indegree, cross-layer outdegree [33], and multiplex PageRank [34] to identify influential malicious accounts across different layers of the ACMN. Details of the metrics for identifying malicious accounts are presented in the following paragraphs.

- Indegree:The indegree $d_{in}^{l}\left(v\right)$ of node *v* in layer *l* is the number of links that point to *v*. Let $N_{in}^{l}\left(v\right)=\left\{u|\left(u,v\right)\in E^{l}\right\}$ be the in-neighbors of node *v* in layer *l*. The indegree $d_{in}^{l}\left(v\right)$ is calculated by Equation (1).
  (1) $$d_{in}^{l}\left(v\right)=\left|N_{in}^{l}\left(v\right)\right|$$
  A malicious account with a high value of indegree means that its articles have received much attention from other users of the same layer. For example, as shown in Figure 3, $d_{in}^{K}\left(S_{1}\right)=2$, $d_{in}^{K}\left(S_{2}\right)=0$, $d_{in}^{K}\left(S_{3}\right)=3$, $d_{in}^{K}\left(S_{5}\right)=1$, and $d_{in}^{K}\left(S_{6}\right)=1$; thus, the most influential malicious account in layer $G^{K}$ in terms of attracting comments from others is $S_{3}$ as it has the highest indegree in this layer.
  Outdegree: The outdegree $d_{out}^{l}\left(v\right)$ of node *v* in layer *l* is the number of out-going links that point from *v*. Let $N_{out}^{l}\left(v\right)=\left\{u|\left(v,u\right)\in E^{l}\right\}$ be the out-neighbors of node *v* in layer *l*. The outdegree $d_{out}^{l}\left(v\right)$ can be calculated using Equation (2).
  (2) $$d_{out}^{l}\left(v\right)=\left|N_{out}^{l}\left(v\right)\right|$$
  An active malicious account typically joins most of the conversations to influence public opinion; therefore, its outdegree is usually much higher than that of others. For example, as shown in Figure 3, $d_{out}^{K}\left(S_{1}\right)=1$, $d_{out}^{K}\left(S_{2}\right)=2$, $d_{out}^{K}\left(S_{3}\right)=1$, $d_{out}^{K}\left(S_{5}\right)=0$, and $d_{out}^{K}\left(S_{6}\right)=3$. Thus, $S_{6}$ is considered to be the most active account in layer $G^{K}$ in terms of commenting activity since it has commented on the articles of almost all other accounts.
  Cross-indegree: The cross-indegree of node *v* in a multilayer network M can be defined as the number of its unique in-neighbors across the different layers of the network. The cross-indegree $d_{in}^{M}\left(v\right)$ of node *v* in M is defined as in Equation (3).
  (3) $$d_{in}^{M}\left(v\right)=\left|\cup_{l\in M}N_{in}^{l}\left(v\right)\right|$$
  where $N_{in}^{l}\left(v\right)$ is a set of in-neighbors of *v* in layer *l*. In Figure 3, $N_{in}^{T}\left(S_{1}\right)=\left\{S_{2}\right\}$, $N_{in}^{H}\left(S_{1}\right)=\left\{S_{2}\right\}$, and $N_{in}^{K}\left(S_{1}\right)=\left\{S_{2},S_{6}\right\}$, thus, $d_{in}^{M}\left(S_{1}\right)=2$. The cross-indegree allows us to evaluate the importance of a node through different layers of the ACMN in terms of attracting attention from other users for its published articles.
  Cross-outdegree: Similar to the cross-indegree, the cross-outdegree of node *v* in a multilayer network M is the total number of its unique out-neighbors over all layers of the network. The cross-outdegree $d_{out}^{M}\left(v\right)$ of node *v* in M can be calculated using the following equation:
  (4) $$d_{out}^{M}\left(v\right)=\left|\cup_{l\in M}N_{out}^{l}\left(v\right)\right|$$
  where $N_{out}^{l}\left(v\right)$ is a set of out-neighbors of *v* in layer *l*. For example, as shown in Figure 3, $N_{out}^{T}\left(S_{1}\right)=\left\{S_{3},S_{4}\right\}$, $N_{out}^{H}\left(S_{1}\right)=\left\{S_{4}\right\}$, and $N_{out}^{K}\left(S_{1}\right)=\left\{S_{3}\right\}$, thus, $d_{out}^{M}\left(S_{1}\right)=2$. The cross-outdegree is a way to measure the activeness of users in terms of commenting across the different layers of the ACMN. The higher cross-outdegree the node has, the more active in commenting on articles posted by others it is.
  PageRank: PageRank is a well-known algorithm for ranking web pages for Google search engine [35]. The PageRank of a page makes contributions to the pages that it points to. In other words, a page receives a high rank if the pages that point to it have high ranks. In this paper, we adopted PageRank for ranking malicious accounts in a single layer as we consider that a malicious account has a high influence on the network if it receives comments from other highly influential users. The PageRank of node *v* in layer *l* of a multilayer network M is computed as in Equation (5).
  (5) $$P^{l}\left(v\right)=d\sum_{u\in N_{in}^{l}\left(v\right)}\frac{P^{l}\left(u\right)}{d_{out}^{l}\left(u\right)}+\frac{\left(1-d\right)}{n^{l}}$$
  In Equation (5), $n^{l}$ is the number of nodes in layer *l*, $N_{in}^{l}\left(v\right)$ is a set of in-neighbors of *v*, $d_{out}^{l}\left(u\right)$ is the outdegree of *u*, and *d* is the damping factor which is generally set as 0.85 [35].
  Multiplex PageRank: As aforementioned, PageRank is used to find influential nodes in a single-layer network. However, it cannot be used to rank nodes in a multilayer network. Multiplex PageRank, an extension of PageRank, was proposed to rank nodes in multiplex networks [34]. In this paper, we suppose that the centrality of a malicious account in a layer influences its centrality in other layers. Therefore, additive multiplex PageRank was adopted to rank malicious accounts in the ACMN [36]. Generally, additive multiplex PageRank is similar to the original PageRank [35]; however, in additive multiplex PageRank, when calculating the PageRank centrality of a node in one layer, its PageRank centrality in other layers is considered. The additive multiplex PageRank of nodes in layer β with respect to layer γ is calculated by adding some values to the weight of PageRank the nodes have in layer β in proportion to the weight of PageRank that they have in layer γ. Mathematically, the additive Multiplex PageRank of node *v* in layer β with respect to layer γ, denoted as $P^{\beta }\left(v\right)$, is calculated according to in Equation (6).
  (6) $$P^{\beta }\left(v\right)=d\sum_{u\in N_{in}^{\beta }\left(v\right)}\frac{P^{\beta }\left(u\right)}{d_{out}^{\beta }\left(u\right)}+\left(1-d\right)\frac{P^{\gamma }\left(v\right)}{N<P^{\gamma }>}$$
  where $P^{\gamma }\left(v\right)$ denotes the PageRank of node *v* in layer γ, $<P^{\gamma }>$ is the average of PageRank of *N* nodes in layer γ, and *d* is the damping factor which is also set around 0.85.
  The process of calculating the PageRank of nodes in the ACMN is described as Algorithm 1.

**Algorithm 1** Calculating the PageRank of nodes in the ACMN.
**Input:**
$M=\left\{G^{T},G^{H},G^{K}\right\}$
**Output:** Multiplex PageRank of nodes in M1: Calculate the largest eigenvalue of each layer.2: Arrange the layers in descending order of their eigenvalues as a larger eigenvalue indicates faster information dissemination [37].3: Calculate the PageRank of the 1st layer using Equation (5).4: Calculate the PageRank of the 2nd layer with respect to the 1st layer using Equation (6).5: Calculate the PageRank of the 3rd layer with respect to the 2nd layer according to Equation (6).6: **Return:** The PageRank of the 3rd layer is the PageRank of the ACMN.

#### 3.4.2. Malicious Account Influence Across Layers (RQ1.3)

The activeness of malicious accounts may vary across different layers of the ACMN; for example, some malicious accounts are probably very active in commenting and posting articles related to Ko, but they rarely comment and write articles talking about Tsai and Han. On the other hand, other malicious accounts may be active in all discussions related to the three candidates. Therefore, we are interested in verifying whether the activeness of malicious users is persistent across different layers of the network. Thus, for addressing RQ1.3, Spearman’s rank correlation coefficient, which has been widely used to evaluate the similarity between two ranked sets [29,38], was used to evaluate the pairwise correlations of the ranking orders of layers in terms of indegree, outdegree, and PageRank. The Spearman’s rank correlation coefficient between layers β and γ, denoted as $\rho^{\beta ,\gamma }$, is calculated using Equation (7).

(7) $$\rho^{\beta ,\gamma }=1-\frac{6\sum_{v\in V}d_{v}^{2}}{N(N^{2}-1)}$$

where $d_{v}$ is the difference of the ranks of node *v* in layers β and γ, and *N* is the total number of nodes that appear in all layers of the ACMN. The value of $\rho^{\beta ,\gamma }$ is in the range [−1,1]. The closer the value of $\rho^{\beta ,\gamma }$ to 1 or −1, the stronger positive or negative correlations between the ranks of nodes in layers β and γ. When $\rho^{\beta ,\gamma }=0$, there is no correlation between two ranked sets.

### 3.5. Node Similarity (RQ2)

In addition to identifying the influential malicious accounts in the ACMN, another goal of this study is to examine whether the interactions between malicious accounts are persistent across different layers. Inspired by Zhang and Ye [39], we compute the node similarity in two cases: indegree and outdegree. The node indegree similarity measures the consistency of users in terms of attracting comments. The node outdegree similarity is used to verify whether the commenting behavior of a user is consistent across different layers.

Let $A^{l}=\left\{a_{v,u}^{l}\right\}$ be the adjacency matrix of layer *l*, where $a_{v,u}^{l}$ denotes the weight of the link from *v* to *u* of layer *l*. Let $w_{vu}^{l}$ be the number of comments *v* has given to articles posted by *u*. An element $a_{v,u}^{l}$ is expressed as follows:

(8) $$a_{vu}^{l}=\left\{\begin{array}{cc} w_{vu}^{l} & \mathrm{if}\;\mathrm{there}\;\mathrm{is}\;a\;\mathrm{directed}\;\mathrm{link}\;\mathrm{from}\;v\;\mathrm{to}\;u\;\mathrm{of}\;\mathrm{weight}\;w_{vu}^{l}\\ 0, & \mathrm{otherwise} \end{array}\right.$$

The relationship between node *v* and other nodes in layer *l* regarding the indegree and outdegree can be represented as vectors $R_{in}^{l}\left(v\right)$ and $R_{out}^{l}\left(v\right)$ of *N* elements, respectively, each of which is expressed as follows:

(9) $$R_{in}^{l}\left(v\right)=\left[a_{1v}^{l},a_{2v}^{l},a_{3v}^{l},\ldots ,a_{Nv}^{l}\right]$$

(10) $$R_{out}^{l}\left(v\right)=\left[a_{v1}^{l},a_{v2}^{l},a_{v3}^{l},\ldots ,a_{vN}^{l}\right]$$

The similarity of node *v* between layers β and γ based on indegree can be considered to be the cosine similarity between vectors $R_{in}^{\beta }\left(v\right)$ and $R_{in}^{\gamma }\left(v\right)$. Please note that we only consider the nodes that appear in both layers β and γ for calculating the node similarities. Therefore, the number of elements in vectors $R_{in}^{\beta }\left(v\right)$ and $R_{in}^{\gamma }\left(v\right)$ is the same. The same can be done for node similarity based on outdegree. The similarities of node *v* between layers β and γ in terms of indegree, denoted as $SC_{in}^{\beta ,\gamma }\left(v\right)$, and outdegree, denoted as $SC_{out}^{\beta ,\gamma }\left(v\right)$, are defined in Equations (11) and (12), respectively.

(11) $$SC_{in}^{\beta ,\gamma }\left(v\right)=\frac{R_{in}^{\beta }\left(v\right)R_{in}^{\gamma }\left(v\right)}{|R_{in}^{\beta }\left(v\right)||R_{in}^{\gamma }\left(v\right)|}$$

(12) $$SC_{out}^{\beta ,\gamma }\left(v\right)=\frac{R_{out}^{\beta }\left(v\right)R_{out}^{\gamma }\left(v\right)}{|R_{out}^{\beta }\left(v\right)||R_{out}^{\gamma }\left(v\right)|}$$

### 3.6. Community Analysis (RQ3)

To address RQ3, we first apply Louvain community detection algorithm to extract the communities of each layer of the ACMN. We then select the prominent communities of each layer based on their populations. Finally, we identify the most influential malicious accounts in each prominent community according to their indegree and outdegree.

#### 3.6.1. Community Structure

On social networks, a community is a group of users with similar interests, sharing similar content, and interacting with other users in the same group rather than those of other groups [40]. Mining the community structure of the ACMN helps us to understand the latent patterns of malicious accounts within their community. To discover the community structure of each layer in the ACMN, we limit the nodes to those who are malicious users. We apply a well-known community detection algorithm, namely Louvain, proposed by Blondel et al. [41] to extract the communities in each layer of the network. This algorithm consists of two steps. First, each node is considered to be a community itself. Then, for each node, the gain of modularity is evaluated, and the node is placed into one of its neighbors’ communities if moving it to this community the gain in modularity is maximize and positive; otherwise it remains in its original community. This process is applied repeatedly and stopped when the modularity is no further increase.

#### 3.6.2. Prominent Community (RQ3.1)

To address RQ3.1, we need to identify the communities that have a significant number of nodes compared to other communities of the same layer. Let $n^{l}$ and $nc^{l}$ be the numbers of nodes and communities in layer *l*, respectively, and let $n_{c}^{l}$ denotes the number of nodes in community *c* of layer *l*. The community *c* of layer *l* is labeled as a prominent community according to the following equation:

(13) $$PC^{l}\left(c\right)=\left\{\begin{array}{cc} true & \mathrm{if}\;\left(n_{c}^{l}\geq \left(\frac{n^{l}}{nc^{l}}\right)\theta \right)\\ false, & \mathrm{otherwise} \end{array}\right.$$

where θ is the adjustment parameter; the bigger this parameter, the more prominent the community is.

#### 3.6.3. Opinion Leaders of Prominent Community (RQ3.2)

For each layer, we are also interested in finding the malicious accounts that are the most influential in each prominent community. This helps us to figure out which accounts are the opinion leaders of each community as well as of each discussion network. Let $K_{ol}^{l}$ be the number of desired opinion leaders that we need to identify for layer *l*. The top opinion leaders $Tk_{ol}^{l}\left(c\right)$ of community *c* to be identified is defined as in Equation (14).

(14) $$Tk_{ol}^{l}\left(c\right)=\left\lceil K_{ol}^{l}*\frac{n_{c}^{l}}{\sum_{i=1}^{pc^{l}}n_{i}^{l}}\right\rceil |PC^{l}\left(c\right)=PC^{l}\left(i\right)=true$$

where $pc^{l}$ and $n_{i}^{l}$ are the number of prominent communities and the number of nodes of prominent community *i* in layer *l*, respectively.

When the number of opinion leaders to be identified for each prominent community is specified, we select the top Tk opinion leaders for each prominent community based on their indegree and outdegree.

### 3.7. Community Behavior (RQ4)

To answer RQ4, we compute the number of articles and the number of comments posted by each community in the network. We also calculate the daily number of articles and the daily number of comments posted by each community during our observation timeframe. The computed results are then investigated to find the communities with apparent behavior differences from others.

#### 3.7.1. Thread Starting Community and Active Commenting Community (RQ4.1)

To answer RQ4.1, we focus on investigating the communities that are active in each layer of the ACMN in terms of commenting and posting articles. We calculate the total number of comments and the total number of articles posted by each community. We consider a community that posts a majority of articles as the thread starting community (TSC). Similarly, a community that gives a lot of comments is called the active commenting community (ACC). Labeling a community can be formulated as follows:

- TSC: Let $PA_{c}^{l}$ denotes the number of articles posted by community *c* of layer *l*, and $PA^{l}$ be the total number of articles posted by all users in layer *l*. The community *c* of layer *l* is labeled as the TSC using the following equation:
  (15) $$TSC^{l}\left(c\right)=\left\{\begin{array}{cc} true & \mathrm{if}\;\left(PA_{c}^{l}\geq \left(n_{c}^{l}\frac{PA^{l}}{n^{l}}\right)\theta \right)\\ false, & \mathrm{otherwise} \end{array}\right.$$
  where $n_{c}^{l}$ and $n^{l}$ are the number of nodes in community *c* of layer *l* and the total number of nodes in layer *l*, respectively. θ is the adjustment parameter; the bigger this parameter, the more active in posting articles the community is.
- ACC: Let $PC_{c}^{l}$ be the number of comments posted by community *c* of layer *l*, and $PC^{l}$ be the total number of comments posted by all users in layer *l*. The community *c* is labeled as the ACC according to the following expression:
  (16) $$ACC^{l}\left(c\right)=\left\{\begin{array}{cc} true & \mathrm{if}\;\left(PC_{c}^{l}\geq \left(n_{c}^{l}\frac{PC^{l}}{n^{l}}\right)\theta \right)\\ false, & \mathrm{otherwise} \end{array}\right.$$
  where $n_{c}^{l}$ and $n^{l}$ are the number of nodes in community *c* of layer *l* and the total number of nodes in layer *l*, respectively. θ is the adjustment parameter; the bigger this parameter, the more active in commenting the community is.

#### 3.7.2. Temporal Pattern (RQ4.2)

To address RQ4.2, we analyze the temporal patterns of commenting and posting articles of each community in the ACMN by calculating the daily number of articles and the daily number of comments publishing by the malicious accounts during our observation timeframe. The calculated results are further explored to find the communities with apparent behavior differences from others.

## 4. Results and Discussion

In this section, we present the experimental results using our approach as described in Section 3 and discuss our findings.

### 4.1. Characteristics of the ACMN

The ACMN was constructed according to the method described in Section 3.3. Table 2 shows the properties of the network in details. It is noted that the $G^{T}$, $G^{H}$, and $G^{K}$ layers represented the discussions related to candidates Tsai Ing-Wen, Han Kuo-Yu, and Wen Je-Ko, respectively. In the table, edges labeled as malicious-malicious indicate the interaction between malicious users and edges that denoted as malicious-normal stand for the interaction between malicious and normal users. It can be seen from Table 2 that the discussions related to Han received more attention from normal users (40,057) compared to those of Tsai (33,694) and Ko (32,404). Additionally, we computed the graph density of each layer of the ACMN. The density d(G) of a directed graph G(V,E) is the number of edges divided by the number of possible edges. The density, d(G), is $d\left(G\right)=\frac{\left|E\right|}{\left|V|(|V|-1\right)}$, where |E| is the number of edges and |V| is the number of vertices [42]. The density d(G) ranges from 0, if there is no edge, to 1, if all edges are present. The density of a graph is used to measure how connected it be compared to how connected it could be. It can be seen from Table 2 that the network density of $G^{H}$ was higher than that of $G^{T}$ and $G^{K}$, which indicates that there was more interactions between users in Han’s discussion compared to the other two candidates.

**Figure 4:**
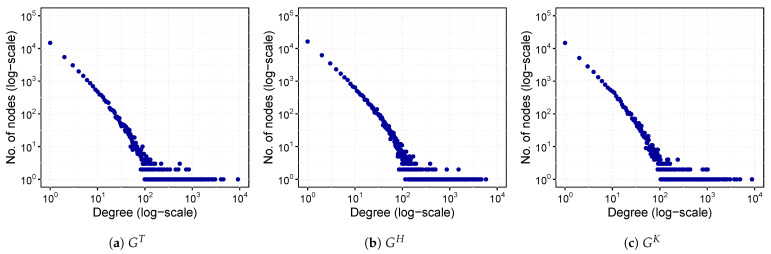
Degree distribution of layers of the ACMN in log-scale.

Furthermore, we are also interested in finding whether the degree distribution of nodes in each layer of the ACMN follows the power-law distribution. A layer is considered to be a power-law network when the probability distribution of degree *d*, p(d), follows a power-law $p\left(d\right)\propto d^{\delta }$, where δ≥1 is an exponential parameter of the power-law distribution and its value is around 2 [43]. Figure 4 presents the histogram of the degree distribution of each layer of the ACMN in log-scale. It can be seen from the graph that the degrees of nodes in the three layers obey a power-law distribution. This indicates that some malicious accounts have attracted many other users for commenting on their articles, and they were also active in commenting on the articles posted by other users, while the majority of other accounts being less active. To estimate the δ parameter, we used the maximum likelihood approach proposed by [44]. This method uses the Kolmogorov-Smirnov test for calculating the goodness of fit [45]. We obtained δ=2.65 with p=0.636 for $G^{T}$, δ=1.94 with p=0.083 for $G^{H}$, and δ=1.96 with p=0.150 for $G^{K}$. As the values of δ are around 2 and the *p* values are greater than 0.05, we can confirm that the data follows the power-law distribution and the three layers can be considered to be scale-free networks [46].

### 4.2. Influential Malicious Accounts (RQ1)

#### 4.2.1. Influential Malicious Accounts of a Single-Layer (RQ1.1)

**Figure 5:**
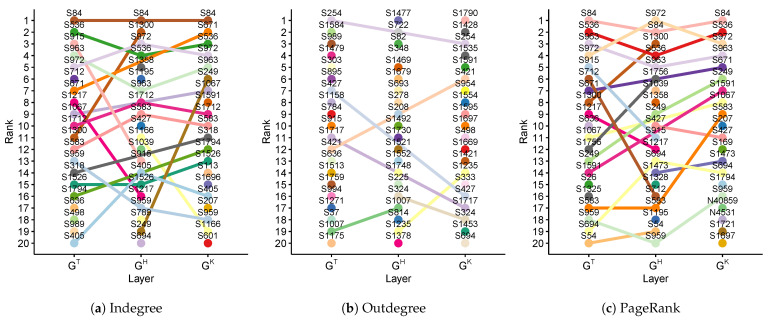
Ranks of the top 20 malicious accounts based on indegree, outdegree, and PageRank of each layer of the ACMN.

First, we identified influential malicious accounts that received comments from the majority of users of each layer of the ACMN. These accounts can be considered to be nodes with high values of indegree. Figure 5a shows the top 20 nodes ranked according to their indegree for the three layers. As shown in the figure, most of the users on the top 20 in one layer also appeared in the top 20 of other layers. For instance, user S84 was ranked 1st in all layers $d_{in}^{T}=687$, $d_{in}^{H}=721$, $d_{in}^{K}=835$), which may imply that the articles posted by this user attracted a lot of attention from other users in the political discussion on PTT. Similarly, users S536, S963, S972, and S1712 were ranked in the top 10 of all layers. In contrast, some users appeared in the top 20 of one layer only. For instance, user S712 was ranked in the top 20 of $G^{T}$ but not in $G^{H}$ and $G^{K}$ (6th-$G^{T}$, 31st-$G^{H}$, 48th-$G^{K}$). In particular, we found that the rank of user S1195 is high in $G^{H}$ but extremely low in $G^{T}$ and $G^{K}$ (6th-$G^{H}$, 362nd-$G^{T}$, 436th-$G^{K}$).

Regarding the malicious accounts that give comments to many other users, we ranked the users of each layer by their outdegree. The top 20 malicious accounts ranked by their outdegree are presented in Figure 5b. From the figure, we can see that the majority of the top 20 users only appear in one layer. For instance, users S1477 and S1790 were ranked 1st in $G^{H}$ and $G^{K}$, respectively; however, their ranks in other layers were low (15,222nd-$G^{T}$, 63rd-$G^{K}$ for S1477; and 34th-$G^{T}$, 115th-$G^{H}$ for S1790). Moreover, a few malicious accounts were in the top 20 of two layers but did not appear in the remaining layer; for instance, user S254 was ranked 1st in $G^{T}$ and 3rd in $G^{K}$ but 30th in $G^{H}$.

Figure 5c shows the top 20 users based on PageRank. For PageRank, users have high PageRank scores if they receive comments from other users who also get comments from many others. These users are considered to be important nodes in their own network [47]. According to the results, four influential malicious accounts were ranked on the top 5 of all layers (i.e., S84, S536, S963, and S972). Some malicious accounts were highly ranked in one layer only; for example, S636 ranked 10th-$G^{T}$, 55th-$G^{H}$, and 45th-$G^{K}$; S1039 ranked 25th-$G^{T}$, 7th-$G^{H}$, and 91st-$G^{K}$.

It is evident from the above finding that there were several malicious accounts that have attracted comments from a lot of users in all discussions related to the three candidates, while a few accounts only get attention from the users in the discussion related to one candidate only. It is also found that there were some malicious accounts that were very active in giving comments in the discussion related to their focusing candidate while being less active in the discussions related to other candidates. From the aforementioned analysis, the top 20 accounts depicted in Figure 5 are considered to be the influential malicious users, in terms of a single-layer, to answer RQ1.1.

#### 4.2.2. Influential Malicious Accounts of the ACMN (RQ1.2)

The indegree, outdegree, and PageRank measures can only work with single-layer networks; thus, we introduced the cross-layer measures to find the important nodes across layers of the ACMN, as presented in Section 3.4. For calculating the multiplex PageRank for nodes of the ACMN, we ordered the layers by their maximum eigenvalues. The maximum eigenvalues were 0.616, 0.386, and 0.587 for $G^{T}$, $G^{H}$, and $G^{K}$, respectively. Therefore, the ordering of the layers for calculating multiplex PageRank was $G^{T}$, $G^{K}$, and $G^{H}$. In Table 3, we report the top 20 influential malicious accounts ranked by cross-indegree, cross-outdegree, and multiplex PageRank. From the table, S254 was the most active malicious users in terms of giving comments to other users ($d_{out}^{M}=$ 14,076), followed by S1790 ($d_{out}^{M}=9974$). The cross-outdegree scores of these two accounts were much higher compared to others. Regarding the malicious accounts that received comments from many users in the ACMN, S84 was ranked 1st ($d_{in}^{M}=2204$), followed by S536 ($d_{in}^{M}\;=\;1840$); and the number of users commented on their articles was considerably bigger than that of other highly ranked users. Regarding the malicious accounts that received comments from other highly important users, the multiplex PageRank scores of accounts S536, S84, and S972 were relatively high compared to other accounts. The malicious accounts shown in Table 3 are the users we needed to identify to address RQ1.2. These accounts are considered to be the most influential malicious accounts across layers of the ACMN.

#### 4.2.3. Malicious Account Influence Across Layers (RQ1.3)

To verify whether the ranks of malicious users across different layers of the ACMN are persistent, we calculated the Spearman’s rank correlation coefficient of indegree, outdegree, and PageRank for each pair of layers according to Equation (7). The results are shown in Table 4. From the table, we observed that the ranking based on indegree has a high correlation among the three layers of the ACMN. In particular, the Spearman’s rank correlation of indegree between layers $G^{T}$ and $G^{H}$ was 0.84 (p-value<0.01), which indicates that the ranks of malicious accounts based on indegree did not vary among the discussions related to Tsai and Han. In other words, the influence of malicious users, in terms of attracting comments from other users on their articles related to Tsai and Han, was highly associated. Regarding the commenting activity, the correlation of outdegree across the layers was relatively weak, with the Spearman’s rank correlation coefficient of around 0.6. This may imply that malicious users tend to comment on articles related to their focusing candidates only. The PageRank correlations between the layers of ACMN was also high (i.e., $\rho^{G^{T},G^{H}}=0.70$, $\rho^{G^{T},G^{K}}=0.71$, and $\rho^{G^{H},G^{K}}=0.65$). This implies that influential malicious accounts that got comments from important users in one layer also received comments from influential users in other layers. The above analysis is for addressing RQ1.3 that the influence of malicious accounts across different layers was highly persistent in terms of attracting comments but varied in terms of giving comments.

### 4.3. Node Similarity (RQ2)

We computed the node indegree and outdegree similarities of the top 20 malicious accounts according to Equations (11) and (12), respectively. The results are shown in Table 5. A node with a high similarity score indicates that the interaction between this node and its neighbors is more stable compared to other nodes for each pair of layers. For instance, regarding the node indegree similarity, user S84, the most influential malicious account in terms of attracting comments, tended to receive an approximate number of comments from the same set of users related to Tsai’s and Ko’s discussions ($SC_{in}^{T,K}=0.435$). Similarly, account S1300 received relatively stable attention from other users among the discussions related to Tsai and Han ($SC_{in}^{T,H}=0.424$). Regarding the node outdegree similarity, the most active malicious account, S254, tended to give comments to the same set of users in layers $G^{T}$ and $G^{H}$ ($SC_{out}^{T,H}=0.324$). Likewise, other users in the top 10 influential malicious users have also given approximate numbers of comments to the same users in Tsai’s and Han’s discussions (i.e., S1469, S1595, S324). Accounts S1584 and S1477 only commented on the articles related to their focusing candidates. S1584 focused the discussion related to Tsai and Han while S1477 favored Han’s and Ko’s discussions. Overall, we find that a few influential malicious accounts had relatively consistent interactions with other accounts across different layers while most of others did not. This finding is for resolving RQ2.

### 4.4. Community Analysis (RQ3)

To investigate the community structure of each layer of the ACMN, we used Gephi (https://gephi.org (accessed on 15 September 2020)), an open-source software for network visualization and exploration, to construct the networks. We aim to assess the interactions between malicious accounts; thus, we limited the nodes to those who are malicious users. The Louvain community detection algorithm was deployed to extract the communities in each layer. This algorithm resulted in 8, 8, and 11 communities with the modularity values of 0.326, 0.375, and 0.408 for $G^{T}$, $G^{H}$, and $G^{K}$, respectively; the higher the modularity value, the stronger the information dissemination strength.

#### 4.4.1. Prominent Community (RQ3.1)

**Figure 6:**
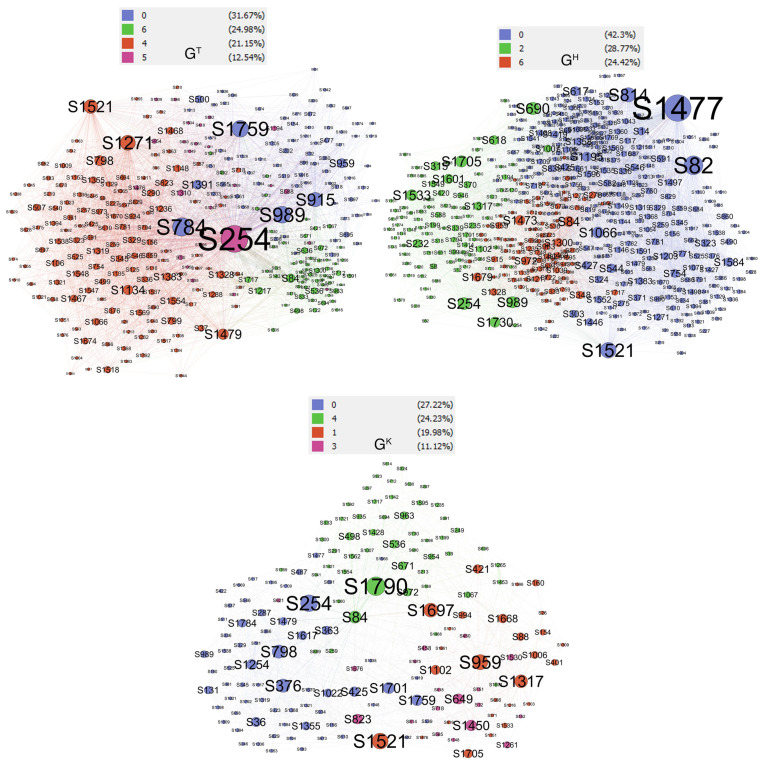
Prominent communities of each layer of the ACMN.

The prominent communities of the ACMN were identified according to Equation (13) (θ=1). Figure 6 shows the prominent communities for each layer of the network. For better visualization, nodes whose degrees were less than 10 were filtered from the graphs. The table on the top of each graph shows lists of numbers and colors which were assigned by Gephi to its corresponding communities. The left column of this table shows the percentage of nodes that belong to each community. Please note that the size of a node is proportional to its degree. The prominent communities account for 90.34%, 95.49%, and 82.55% of the nodes of layers $G^{T}$, $G^{H}$, and $G^{K}$, respectively. These communities are the prominent communities we needed to find to respond to RQ3.1.

#### 4.4.2. Opinion Leaders of Prominent Community (RQ3.2)

**Figure 7:**
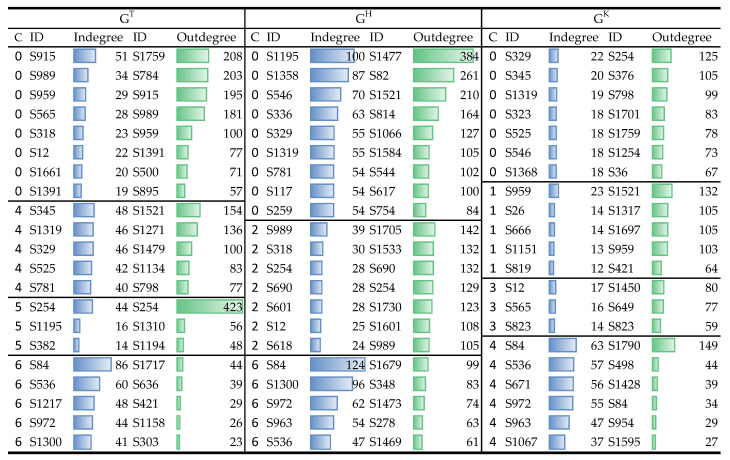
Top opinion leaders of each community of the ACMN.

We further identified the opinion leaders for each community of the ACMN. For the number of desired opinion leader of 20, the top opinion leaders of each community calculated according to Equation (14) are shown in Figure 7. For $G^{T}$, the indegree scores of the opinion leaders of communities 4 and 6 were relatively higher than those of communities 0 and 5, which means that these accounts received more attention from other malicious users compared to other leaders of this layer. The outdegree scores of the leaders of community 6 were low, which indicates that the malicious accounts in this community were less active in commenting. Regarding the community 0 of $G^{H}$ the indegree and outdegree scores of its leaders were higher compared to other communities’ leaders. The communities’ leaders of $G^{K}$ received less attention from other users compared to those of other layers as their indegree scores were small, except the community 4. The indegree scores of community 4 of $G^{K}$ were high which imply that the articles posted by the users of this community were attracted more attention from other malicious accounts compared to other communities of this layer. The opinion leaders shown in Figure 7 are those to answer RQ3.2.

### 4.5. Community Behavior (RQ4)

#### 4.5.1. ACC and TSC of the ACMN (RQ4.1)

To find the most active communities in the ACMN, we computed the number of comments and the number of articles posted by each prominent community. We marked the prominent communities as TSC and ACC according to Equations (15) and (16) (θ=1), respectively; and the results are presented in Table 6. As shown in the table, community 6 was notably active in the discussion related to Tsai, in terms of posting articles and giving comments (i.e., the averages of posted articles and comments per user of 4.6 and 66.3, respectively). Communities 0 and 4 of $G^{T}$ were more likely to comment on articles posted by other users rather than to post articles. Community 6 of $G^{H}$ was very active in posting new articles with an average of posted articles per user of 8.4, which was the highest scores compared to other communities. For $G^{K}$, community 4 was the most active community regarding the number of comments and the number of posted articles in this layer. The average of comments per user of this community was much higher compared to other communities not only in this layer but also the whole network (Avg.comments/user=98.1). From the above analysis, we can conclude that the communities 6-$G^{T}$, 6-$G^{H}$, and 4-$G^{K}$ were the most active communities in the ACMN. The marked communities listed in Table 6 are the communities to answer RQ4.1. We further examine the temporal patterns of these communities in the next section.

#### 4.5.2. Temporal Pattern (RQ4.2)

**Figure 8:**
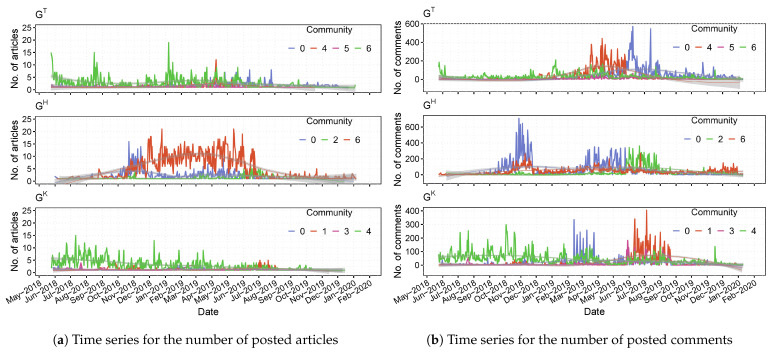
Time series for the number of articles and the number of comments posted by malicious users in each community of the ACMN from 24 May 2018 to 11 January 2020 (the election day).

We present the time series for the number of articles and the number of comments posted by malicious accounts in the prominent communities for each layer of the ACMN in Figure 8.

We can observe from Figure 8a that the most active communities in terms of posting articles, have different temporal trends. For instance, the daily number of articles posted by community 4 of $G^{K}$ gradually decreased during our observation timeframe. The post temporal trend of community 6 of $G^{T}$, which was the most active community in $G^{T}$ regarding the number of posted articles (see Table 6), was relatively stable. In particular, we found that community 6 of $G^{H}$ posted an unusual number of articles from the period of November 2018 to July 2019, which we considered to be the most active community in terms of posting articles in the ACMN. After July 2019, the number of articles posted by this community was significantly low.

Regarding the temporal trends of commenting activity, as shown in Figure 8b, community 0 of $G^{T}$ was not active in commenting from the beginning of our observation timeframe to June 2019, but after that time, the users in this community posted a significant number of comments related to Tsai. Community 4 of $G^{T}$ was very active in commenting from March 2019 to June 2019. The commenting temporal trend of community 4 of $G^{K}$ decreased during the observation timeframe, which was similar to its post temporal trend. We also observed that the commenting temporal trends of communities 0-$G^{T}$, 2-$G^{H}$ and 1-$G^{K}$ were quite similar.

From the above analysis, our findings revealed that the temporal trends of posting articles of prominent communities in the ACMN were different from each other; however, the temporal trends of commenting of a few communities were relatively similar across different layers. These findings are for addressing RQ4.2.

## 5. Conclusions and Future Works

In this study, we proposed an approach to sense influential malicious accounts and investigate their behaviors in a popular online political discussion forum in Taiwan. By modeling the political discussion network as a multilayer network, we extend the understanding of malicious accounts via analyzing different candidates’ discussion network. We addressed four research questions that focused on investigating influential malicious accounts and the communities that they were involved. From the results, we find that several influential malicious accounts received relatively persistent attention from other users across different discussion networks. On the other hand, regarding the commenting activity, the influential malicious users tended to comment on the articles related to their focusing candidates only. The community analysis showed that some communities were very active in commenting and posting new articles while others were only active in commenting activity. Moreover, we find that a few communities posted unusual numbers of articles and comments during special periods of time. To conclude this study, our main contributions are as follows:

- We proposed a new approach for identifying influential users on political discussion networks. In contrast to previous studies that represented an online discussion network as a single-layer network, we modeled the discussion networks as a multilayer network which helped us to investigate user behaviors across different discussion themes.
- We introduced an exploration method to examine the community structure of the discussion network that revealed some communities with unusual commenting and posting articles activities.
- We demonstrated the applicability of the proposed approach by conducting the experiments on the dataset extracted from a political discussion forum during the electoral campaign in Taiwan. According to the experimental results, our method extended the knowledge and understanding of influential malicious accounts with apparent behavior differences from others.

Our approach for studying influential malicious accounts could be beneficial forum moderators and online users to understand the dissemination of information, the ways that the online political discussions are operated, and the roles of these accounts during the election campaigns. Our method can be extended to the electoral campaigns of other countries with different sets of candidates. We hope the study could increase the transparency and democracy of online information. Moreover, our approach can be applied to other online social platforms, regardless of country and language, to explore the roles of influential users and their interactions, especially those accounts that are operated by the same users or groups. The application of the proposed approach is not limited to political discussions, but can also be generalized for applications in other events such as sporting events, promotion campaigns, and epidemic spreading.

From our findings, we cannot be sure that such behavior of influential users are indeed malicious activities. Therefore, the content of comments and articles posted by these accounts needs to be further investigated to verify whether it contains fake news, rumours, or hate speeches. Spreading such malicious content could potentially harm the transparency, fairness, and democracy of social platforms. In the next steps of this study, we aim to propose an identification model via enhanced learning algorithms that leverage content-based features, network-based features, and behavior-based features to detect malicious accounts on the political discussion forums.

## Figures and Tables

**Table 1:** A summary of our dataset.

Sub-Dataset	Candidate	No. of Articles	No. of Comments	No. of Authors	No. of Commenters
1	Tsai Ing-Wen	2193	60,404	347	1365
2	Han Kuo-Yu	3672	73,671	435	1434
3	Ko Wen-Je	2304	67,996	348	1478

**Table 2:** Characteristics of the ACMN.

Layer	No. of Nodes		No. of Edges		Density	Avg. Degree
	Malicious Account	Normal Account	Malicious-Malicious	Malicious-Normal		
$G^{T}$	1441	33,694	6080	175,825	0.000147	10.355
$G^{H}$	1493	40,057	9308	262,318	0.000157	13.075
$G^{K}$	1543	32,404	4319	166,051	0.000148	10.037

**Table 3:** Top 20 influential malicious accounts across layers of the ACMN.

Rank	Cross-Indegree		Cross-Outdegree		Mutiplex PageRank	
	ID	Score	ID	Score	ID	Score
1	S84	2204	S254	14,076	S536	0.00570
2	S536	1840	S1790	9974	S84	0.00553
3	S972	1589	S1584	6628	S972	0.00513
4	S963	1424	S1535	6590	S963	0.00452
5	S671	1247	S1477	6357	S671	0.00387
6	S1300	1144	S989	6271	S249	0.00363
7	S1712	1096	S1469	6239	S1067	0.00217
8	S583	1029	S1595	5886	S1591	0.00207
9	S249	1007	S324	5666	S1473	0.00141
10	S1067	952	S1428	5649	S427	0.00139
11	S915	924	S421	5629	S207	0.00122
12	S1526	848	S1235	5624	S1300	0.00121
13	S318	810	S1521	5604	S694	0.00118
14	S959	800	S1007	5559	S959	0.00117
15	S405	790	S427	5448	S583	0.00108
16	S1794	753	S722	5212	S1712	0.00104
17	S1166	748	S1591	5126	S169	0.00091
18	S1591	725	S1717	5109	S1697	0.00089
19	S1217	721	S1679	5034	S915	0.00086
20	S712	687	S348	4926	S1794	0.00082

**Table 4:** Spearman’s rank correlation between layers for indegree, outdegree, and PageRank.

Layer	Indegree	Outdegree	PageRank
($G^{T}$, $G^{H}$)	0.84 **	0.58 **	0.70 **
($G^{T}$, $G^{K}$)	0.79 **	0.64 **	0.71 **
($G^{H}$, $G^{K}$)	0.73 **	0.56 **	0.65 **

Note: ** Significant at 1% *p* < 0.01.

**Table 5:** Indegree and outdegree similarities of the top 20 influential malicious accounts.

ID	Indegree			Cross-Indegree	ID	Outdegree			Cross-Outdegree
	$\left(G^{H},G^{K}\right)$	$\left(G^{T},G^{H}\right)$	$\left(G^{T},G^{K}\right)$			$\left(G^{H},G^{K}\right)$	$\left(G^{T},G^{H}\right)$	$\left(G^{T},G^{K}\right)$	
S84	0.177	0.242	0.435	2204	S254	0.221	0.324	0.264	14,076
S536	0.268	0.258	0.305	1840	S1790	0.219	0.066	0.141	9974
S972	0.253	0.335	0.290	1589	S1584	-	0.293	-	6628
S963	0.270	0.311	0.334	1424	S1535	0.263	0.132	0.073	6590
S671	0.159	0.129	0.271	1247	S1477	0.254	-	-	6357
S1300	0.156	0.424	0.156	1144	S989	0.138	0.250	0.171	6271
S1712	0.224	0.245	0.218	1096	S1469	0.172	0.447	0.135	6239
S583	0.227	0.211	0.244	1029	S1595	0.250	0.364	0.210	5886
S249	0.222	0.231	0.221	1007	S324	0.226	0.384	0.202	5666
S1067	0.104	0.074	0.192	952	S1428	0.077	0.068	0.172	5649
S915	0.107	0.200	0.087	924	S421	0.168	0.194	0.262	5629
S1526	0.218	0.181	0.219	848	S1235	0.244	0.407	0.190	5624
S318	0.189	0.211	0.120	810	S1521	0.143	0.321	0.179	5604
S959	0.146	0.167	0.100	800	S1007	0.173	0.192	0.215	5559
S405	0.222	0.201	0.200	790	S427	0.134	0.184	0.157	5448
S1794	0.187	0.171	0.233	753	S722	0.076	0.307	0.054	5212
S1166	0.162	0.202	0.195	748	S1591	0.157	0.112	0.191	5126
S1591	0.064	0.085	0.054	725	S1717	0.130	0.157	0.157	5109
S1217	0.085	0.113	0.091	721	S1679	0.144	0.289	0.083	5034
S712	0.130	0.144	0.274	687	S348	0.145	0.313	0.048	4926

**Table 6:** ACCs and TSCs of the ACMN.

Layer	C	No. of Users	No. of Articles	Avg. Articles/User	No. of Comments	Avg. Comments/User	ACC	TSC
$G^{T}$	0	331	450	1.4	20,023	60.5	•	
	4	221	198	0.9	15,875	71.8	•	
	5	131	173	1.3	3476	26.5		
	6	261	1195	4.6	17,294	66.3	•	•
$G^{H}$	0	525	749	1.4	32,382	61.7	•	
	2	357	255	0.7	14,877	41.7		
	6	303	2557	8.4	24,513	80.9	•	•
$G^{K}$	0	301	143	0.5	8013	26.6		
	1	221	213	1.0	10,232	46.3		
	3	123	57	0.5	4080	33.2		
	4	268	1415	5.3	26,298	98.1	•	•

## Data Availability

The data underlying this article will be shared on reasonable request to the corresponding author.
